# In Silico Identification of Potential Inhibitors of the SARS-CoV-2 Main Protease among a PubChem Database of Avian Infectious Bronchitis Virus 3CLPro Inhibitors

**DOI:** 10.3390/biom13060956

**Published:** 2023-06-07

**Authors:** Laurent Soulère, Thibaut Barbier, Yves Queneau

**Affiliations:** Univ Lyon, INSA Lyon, Université Claude Bernard Lyon 1, CNRS, CPE-Lyon, ICBMS, UMR 5246, Institut de Chimie et de Biochimie Moléculaires et Supramoléculaires, Bâtiment Lederer, 1 Rue Victor Grignard, F-69622 Villeurbanne, France

**Keywords:** homology modeling, molecular docking, main protease, SARS-CoV-2, PubChem

## Abstract

Remarkable structural homologies between the main proteases of the severe acute respiratory syndrome coronavirus 2 (SARS-CoV-2) and the avian infectious bronchitis virus (IBV) were revealed by comparative amino-acid sequence and 3D structural alignment. Assessing whether reported IBV 3CLPro inhibitors could also interact with SARS-CoV-2 has been undertaken in silico using a PubChem BioAssay database of 388 compounds active on the avian infectious bronchitis virus 3C-like protease. Docking studies of this database on the SARS-CoV-2 protease resulted in the identification of four covalent inhibitors targeting the catalytic cysteine residue and five non-covalent inhibitors for which the binding was further investigated by molecular dynamics (MD) simulations. Predictive ADMET calculations on the nine compounds suggest promising pharmacokinetic properties.

## 1. Introduction

Potential drugs able to fight the coronavirus disease 2019 (COVID-19) are still very scarce [1]. Several compounds have been reported to be active on the spike protein, the RNA-dependent RNA polymerase, the 3C-like (3CLpro, or Main protease, M^Pro^), and papain-like (PLpro) viral proteases [2], and repurposing approaches were also released [3,4,5]. Bioinformatics and molecular docking techniques applied to available databases and XRD protein structures are essential tools for the discovery of new compounds and therapies effective against COVID-19 [6,7,8,9,10]. Among possible targets, the main protease (M^pro^) is the most studied one owing to its crucial role in the virus replication process [11,12,13], and either non-covalent M^pro^ inhibitors [14] or covalent ones targeting the catalytic cysteine residue [15,16,17,18,19,20] have been described.

The purpose of the present study is to contribute to the identification of other potential drugs using bioinformatics tools, namely homology modeling, molecular docking, XRD structures, and available databases of molecules related to two severe acute respiratory syndrome coronavirus (SARS-CoV) main proteases, the avian infectious bronchitis virus (IBV) 3CLPro and the SARS-CoV-2 M^Pro^. The PubChem BioAssay database collects and describes biological screenings and other assays, including the protocols and the structures of active (and inactive) compounds [21,22]. The idea is to start with a PubChem database of inhibitors active against the avian IBV 3CLPro and look for compounds that could also interact with the SARS-CoV-2 M^Pro^. The bioassay AID 1706, deposited in 2009 by The Scripps Research Institute Molecular Screening Center, is part of the project “Summary of probe development efforts to identify inhibitors of the SARS coronavirus 3C-like protease (3CLPro)” from the avian infectious bronchitis virus [23,24,25,26,27]. The collection of 290,726 samples was tested for the inhibition of the peptide cleavage by 3CLPro using a fluorescent peptide. Among the 405 compounds active on the enzyme, 388 have their 3D structures directly retrievable from the database and can be used for further in silico investigations.

The structural similarity between the 3C-like proteases in coronaviruses supports the interest in undertaking docking studies of compounds active on the avian IBV within the active site of the SARS-CoV-2 M^Pro^. With the crystal structures of the SARS-CoV-2 M^Pro^ in complex with a covalent inhibitor [28] and of the IBV main protease [29] being both available, protein structure homology and docking studies of 3CLPro inhibitors to specifically target the SARS-CoV-2 M^Pro^ active site were performed. Both non-covalent and covalent potential inhibitors were considered. Covalent inhibitors are less frequently studied because they involve an electrophilic functional group (reacting with a nucleophilic residue in the target active site), which may increase toxicity; however, recent work has tended to re-examine covalent inhibition [30,31,32]. SARS-CoV-2 M^Pro^ possesses a catalytic cysteine (Cys145) able to form a covalent bond with inhibitors [33].

**Figure 1:**
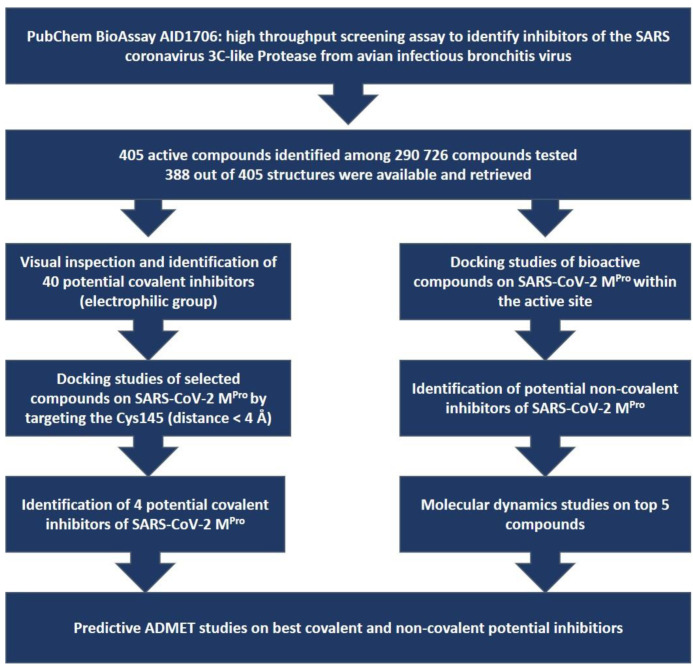
Two approaches to investigating either the covalent or the non-covalent inhibition of the SARS-CoV-2 main protease, starting from the PubChem 3CLPro BioAssay AID 1706.

The assay AID 1706 was retrieved from PubChem BioAssay, and the active compounds available were downloaded as SDF files. Two approaches were then applied: (i) a search for potential covalent inhibitors by selecting, among the starting library of 388 available compounds, those possessing an electrophilic moiety able to form a covalent bond with the Cys145 residue; and (ii) a search for potential non-covalent inhibitors by docking the library within the active site of the SARS-CoV-2 M^Pro^ protein (Figure 1). The stability of the complex of the protein and the potential non-covalent inhibitors was assessed by molecular dynamics (MD), and the predictive ADMET properties were examined.

## 2. Materials and Methods

### 2.1. Homology Studies

The PDB structures of 3C-like proteases of avian IBV (PDB code 2Q6F) [29] and of SARS-CoV-2 (PDB code 6Y2F) [28] were downloaded from the PDB database. The multiple sequence alignment was obtained using the T-COFFEE server [34], with the corresponding FASTA file retrieved from the NCBI database. The ESPript server [35] used with the clustal W file generated the multiple sequence alignment. The PyMOL software generated the 3D-structural alignment between the two proteins and the comparison of the two active sites with divergent amino acids in the surroundings of the catalytic dyad shown in magenta.

### 2.2. Docking Studies

#### 2.2.1. Docking Studies for Covalent Inhibition

The library of compounds active on the main protease of the avian IBV 3CLPro was obtained from the PubChem Bioassay (code AID 1706, https://pubchem.ncbi.nlm.nih.gov/bioassay/1706 (accessed on 5 June 2023)). A set of 388 compounds (out of 405) with their structures fully retrievable were downloaded as an SDF file. This library was then scrutinized to search for compounds bearing a soft electrophilic moiety, resulting in a sub-library of 40 compounds (Table S1, Supplementary Information). Each of them was then docked within the M^Pro^ active site of SARS-CoV-2, centered on the β-keto amide inhibitor, using the Arguslab software v4.0.1 (ArgusLaB 4.0.1; WA planetaria Software LLC: Seattle, WA, USA, 2004) [36,37] with the Argusdock engine and default parameters. The docking results of the 40 compounds were analyzed by focusing on the distance between the sulfur of the cysteine protein active site and the electrophilic center, leading to the selection of four compounds showing a distance <4 Å [16]. The binding modes of these four compounds were examined with PyMOL, and flexible docking experiments were performed using a genetic algorithm engine implemented in Arguslab to corroborate the docking results obtained with the Argusdock engine (Figure S1A, Supplementary Information).

#### 2.2.2. Docking Studies for Non-Covalent Inhibition

Each of the 388 compounds from the SDF library (downloaded from https://pubchem.ncbi.nlm.nih.gov/bioassay/1706 (accessed on 5 June 2023)) were docked within the M^Pro^ active site of SARS-CoV-2, centered on the β-keto amide inhibitor, using Arguslab software v4.0.1 with the Argusdock engine and default parameters. Compounds were then ranked according to their docking score (Table S2), and the corresponding binding modes of the top 5 compounds were examined using PyMOL. The hydrogen bond networks were generated using LigPlot + v2.2.4 [38]. For the five selected compounds, flexible docking experiments with a genetic algorithm engine implemented in Arguslab were performed to corroborate the docking results obtained with the Argusdock engine (Figure S1B).

#### 2.2.3. Molecular Dynamics Studies

The protein-ligand complexes with each of the five best non-covalent potential inhibitors were minimized using the minimization module of MOE 2019 with default parameters. Molecular dynamics simulations were then performed in triplicate on the complexes using the dynamics module of MOE with the AMBER10-EHT force field and R-Field implicit solvation model (dielectric constant ε r = 80), based on the Amber 10 force field for proteins and the Extended Hückel Theory for the ligands [39,40,41]. The Nosé-Poincaré-Andersen (NPA), a sensitive and precise method, was used. The system was equilibrated at 300 K for 50 ps, and 500 ps production was performed, with all other parameters set as default [42,43]. The same study was conducted on the protein alone. Conformational sampling was set up, with each ps leading to 500 frames (Figure S2). Further calculations were performed with the non-covalent inhibitors for 5 ns (NPA method, equilibration at 300 K for 100 ps and 5000 ps production). Conformational sampling was set up with each 10 ps, leading to 500 frames. Trajectories were visualized with VMD, and Calpha RMSD (root-mean-square deviation) fluctuations were analyzed. Conformational samples from each 1000 ps were superimposed to provide an overview of the binding modes and identify the important amino acids bound to each ligand through hydrogen bonds. The hydrogen bond networks, with the distances to each atom, were generated and examined using LigPlot + v2.2.4 [38]. Conformational samples, i.e., protein-ligand complexes from each 1000 ps, were retrieved and submitted as pdb files to the Prodigy webserver to evaluate ΔG_Binding_ [44,45]. The consistency of the method was assessed using the weakest binder, CID 2057165 (Table S2).

## 3. Results and Discussion

### 3.1. Homology Studies

**Figure 2:**
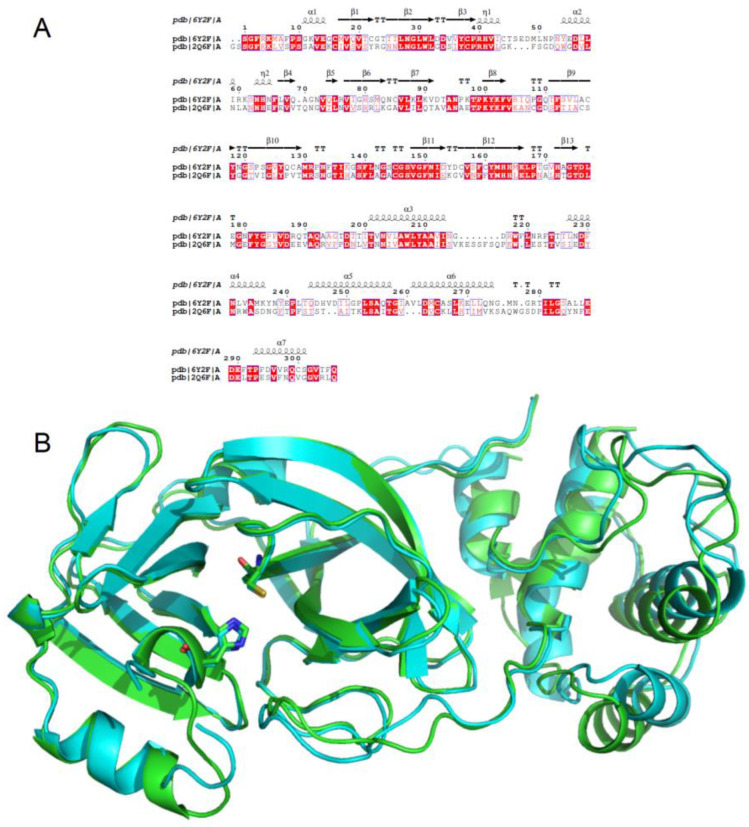
(**A**) Sequence alignment of the two main proteases from the avian IBV and SARS-CoV-2. The conserved residues are framed and highlighted in red (fully conserved) or written in red (partially conserved). The figure was generated using ESPript 3.0 [35]. The secondary structure of the main protease of SARS-CoV-2 is also indicated. (**B**) 3D structural alignment of the two proteins (Calpha RMSD value 1.770 Å) with secondary structure showing the catalytic dyad for both proteins (the SARS-CoV-2 M^Pro^ is colored in cyan).

The structural homology between the main proteases of the avian IBV and of SARS-CoV-2 was investigated by molecular modeling. The amino-acid sequence alignment of the two proteins revealed important similarities and a fully conserved His41 and Cys145 catalytic dyad (SARS-CoV-2 numbering) (Figure 2A) [26].

The 3D structural alignment clearly showed a similar overall secondary structure, with superimposed α-helixes and β-sheets, although some differences in the tertiary structure were observed for the unstructured parts of the two proteins, with a calculated Calpha RMSD value of 1.770 Å (Figure 2B). Importantly, significant superimposition of the residues of the catalytic dyad and an identical 3D structure of the catalytic domain are observed.

**Figure 3:**
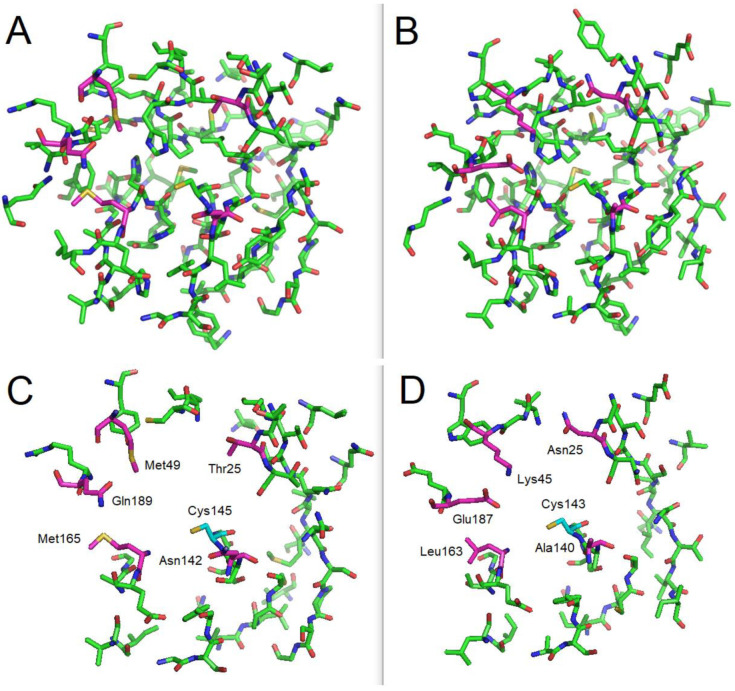
Comparison between the main protease active site of SARS-CoV-2 (**A**,**C**) and avian IBV (**B**,**D**). (**A**,**B**): entire active sites; and (**C**,**D**): active sites simplified by hiding the residues common to the two proteins. The main differences for amino acids close to the catalytic dyad are indicated in magenta.

Although examination of the active sites of the two proteins revealed similarities in the amino acid sequences and conformations, some differences were also identified (Figure 3).

In particular, the histidine 41 environment differs, with Met49, Gln189, Met165, Thr25, and Asn142 for SARS-CoV-2 and Lys45, Glu187, Leu163, Asn25, and Ala140 in IBV. Furthermore, the catalytic cysteine (145 in SARS-CoV-2 or 143 in IBV) is neighbored by the residues Asn142 and Thr25 in SARS-CoV-2 and Ala140 and Asn25 in IBV.

### 3.2. Docking Studies of the 388 Active Compounds from the PubChem BioAssay AID 1706 within the SARS-CoV-2 Protease Active Site

Considering the structural homologies and differences between the two proteins, particularly in their active site, the PubChem BioAssay AID 1706 (IBV) was used as a starting collection to look for potential covalent inhibitors and non-covalent inhibitors directed at the SARS-CoV-2 M^Pro^. The docking method has been validated in our previous study using three covalent inhibitors co-crystallized with SARS-CoV-2 M^Pro^ (PDB codes 5RHF, 5REN, and 5REK) [16], with results consistent with the reported crystallographic data [46].

#### 3.2.1. Docking Studies Looking for Covalent Inhibitors

**Figure 4:**
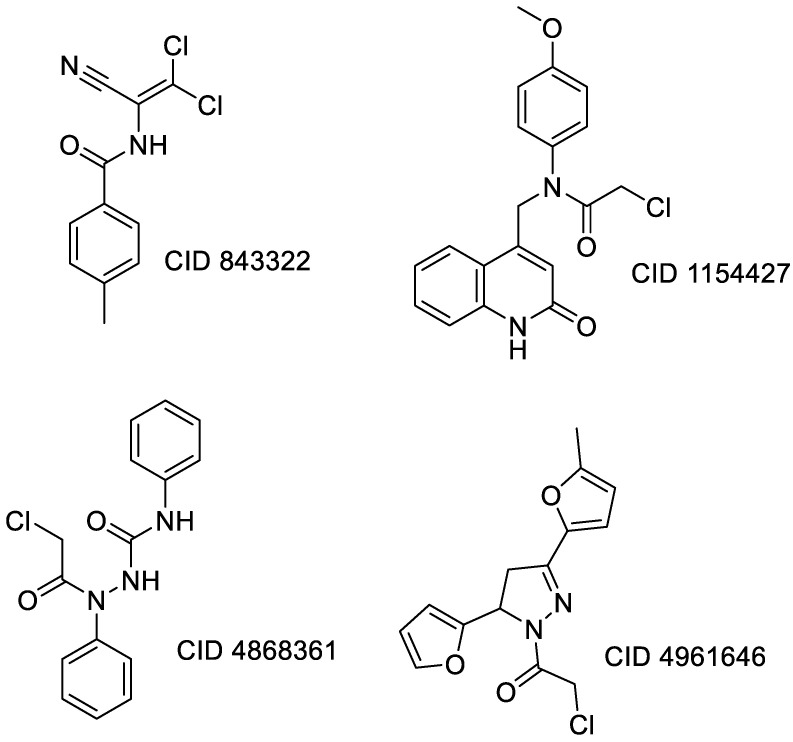
Structure of IBV 3CLPro inhibitors identified as potential covalent inhibitors of SARS-CoV-2 M^Pro^.

Covalent inhibition of SARS-CoV-2 M^Pro^ requires the formation of a covalent bond by the nucleophilic attack of an electrophilic ligand by the thiol group of Cys145 of the active site. Visual inspection of the 388 active compounds present in the bioassay led to the selection of 40 compounds with an electrophilic functional group, including chloroacetyl derivatives, acrylamides, benzonitriles, nitriles, and 2-cyanoacetamides. Docking studies of this sub-library of 40 compounds were analyzed in order to determine whether the distance between the electrophilic residue and the SH group is consistent with the formation of a covalent bond [16]. Four compounds are able, firstly, to bind within the active site of the SARS-CoV-2 M^Pro^ and, secondly, to react with the cysteine residue due to a distance between the sulfur and the electrophilic center of less than 4 Å, have been identified (Figure 4).

Three of the four compounds (CID 1154427, CID 4868361, and CID 4961646) are structurally related to chloroacetamide, while the fourth one is cyanovinyl benzamide (CID 843322 [47]). These compounds interact tightly within the active site with one to four hydrogen bonds, and their electrophilic center clearly lies in the vicinity of the sulfur atom of the cysteine residue (Table 1). The compounds CID 843322 and CID 1154427 interact tightly with the catalytic dyad, while the two other compounds interact with the residues Ser144 and Gly143, in addition to the nucleophilic residue Cys145.

#### 3.2.2. Docking Studies Looking for Non-Covalent Inhibitors

**Figure 5:**
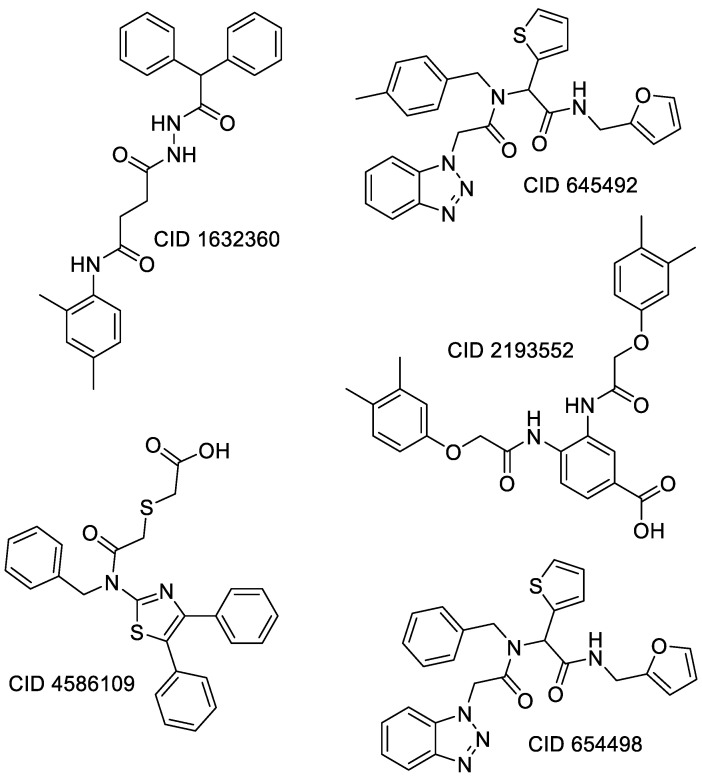
Structure of the five IBV 3CLPro inhibitors identified as non-covalent inhibitors of SARS-CoV-2 M^Pro^.

For the non-covalent inhibition of SARS-CoV-2 M^Pro^, the 388 active compounds on the avian IBV retrieved from the PubChem database were submitted to docking experiments within the active site and ranked according to their calculated docking score (Table S2). The five best binders, with calculated docking scores ranging from −9.92 to −9.54 kcal/mol, are structurally related to hydrazine (CID 1632360), thiazole (CID 4586109), benzotriazole (CID 645492 or CID 654498), or diaminophenyl (CID 2193552) derivatives (Figure 5).

The binding modes of these top five compounds were then studied in more detail. All five interact tightly within the active site, with between one and five H-bonds, and all are located in the close vicinity of the catalytic dyad His41 and Cys145, often interacting with the cysteine residue itself (Table 2).

### 3.3. Molecular Dynamics Studies (Non-Covalent Inhibitors)

**Figure 6:**
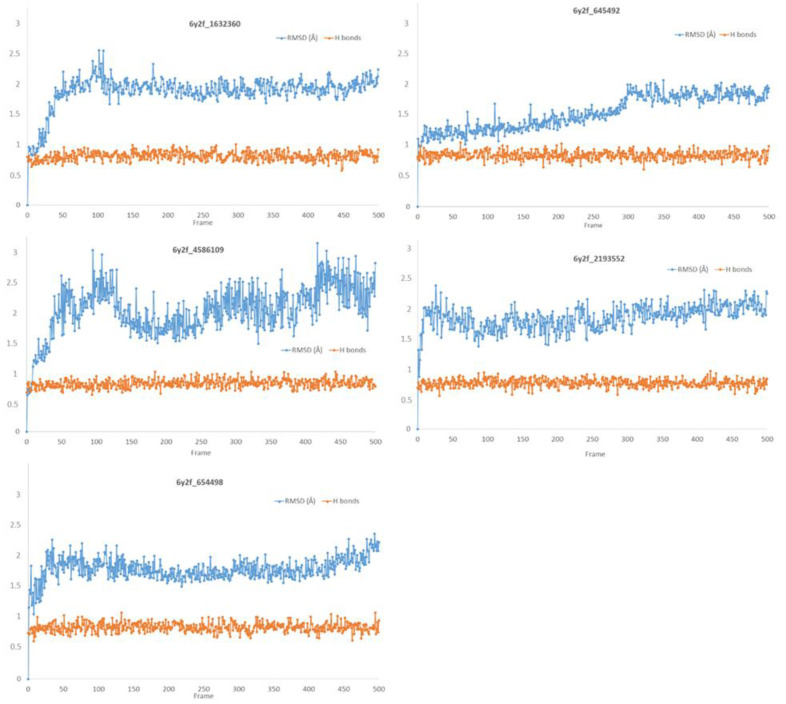
Calpha RMSD obtained by molecular dynamics simulations during 5 ns for all non-covalent complexes (in blue) and H-bonds proportion during the MD simulations calculated as the number of H-bonds per frames divided by the maximum of H-bond number obtained in a given frame (in orange).

The stability of the complexes formed between the protein and the non-covalent potential inhibitors was then investigated by molecular dynamics. First, molecular dynamics simulations were performed for 500 ps in triplicate (Figure S2), showing that the ligands remained tightly bound to the active site over the simulation time. Extension of the simulation time to 5 ns confirmed the stability of the complexes, with Calpha RMSD values fluctuating between 1 Å and 2 Å, except for the compound 4586109 (1.5 Å to 2.5 Å) (Figure 6).

**Figure 7:**
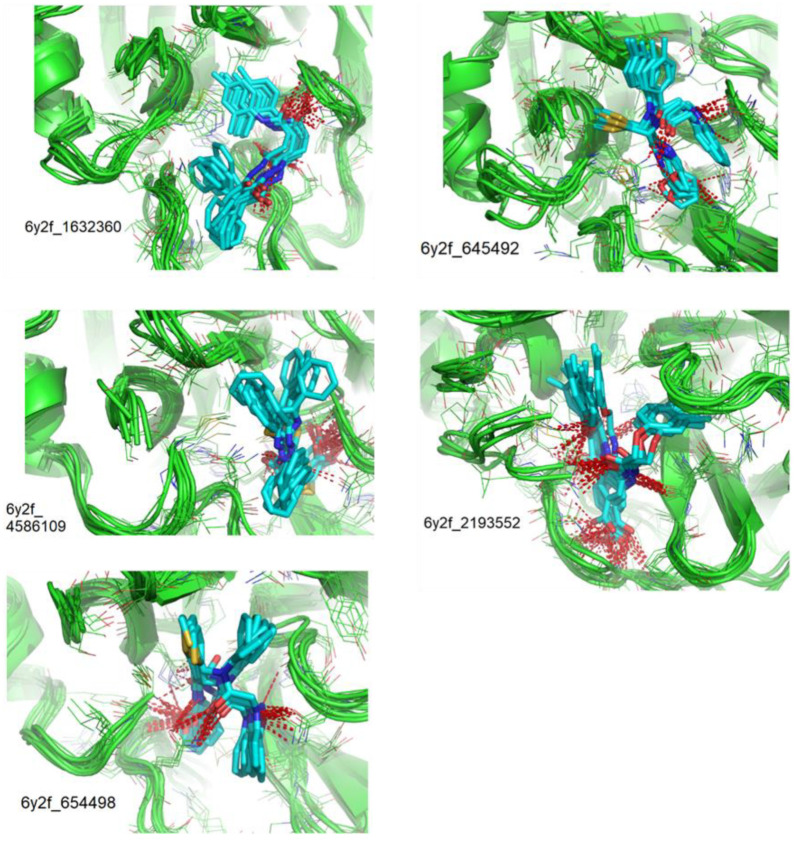
Superimposition of conformational samples collected every 1000 ps for non-covalent inhibitor complexes during molecular dynamics simulations.

The superimposition of conformational samples taken every 1000 ps (six representative samples over the 500 generated) indicates that the different compounds remain bound to the active site during the simulation (Figure 7). To identify the amino acids involved in the interaction with the ligands via hydrogen bonds, six representative samples were examined for each compound using LigPlot+ (European Bioinformatics Institute, Wellcome Trust Genome Campus, Hinxton, Cambridge, UK) [38] (Table 3). The number of residues interacting with the ligand (maximal interactions with five residues at the same time) ranges from three to eight. Some residues are involved in the binding of all five ligands, notably Glu166, already found to be a key residue in other bioinformatic studies [48], and His41 of the catalytic dyad with compound 2193552. Calculations of the RMSD of the ligands also show fluctuations of four ligands (all except for CID 4586109) within the 1 to 2 Å range, supporting good stability of the active site—ligand complexes. For CID 4586109, both protein and ligand RMSD calculations consistently suggest significantly lower complex stability. 

In Figure 6, there are also plotted the ratios between the number of H-bonds over the maximum number of possible H-bonds (both values obtained using the H-bond plugin of VMD) for each ligand, which show remarkable persistence of the H-bond network contribution to the stability of the complexes.

The protein-ligand binding free energies and the H-bond networks during the MD simulation were further investigated by evaluating the ΔG_Binding_ of the complexes collected each 1000 ps and by visualization of these complexes (Table 4).

The ΔG_Binding_ values were evaluated using the Prodigy webserver for protein-ligand complexes [44,45]. The values ranging from −10.63 to −8.7 kcal/mol indicate strong affinities. and are consistent with the docking simulations (see Table 2). For comparison, the binding free energy value for the weakest binder CID 2057165 of the study (Table S2) calculated by the same method is consistently much higher, with a ΔG_Binding_ value of −5.05 kcal/mol. The compound 2193552 develops several interactions with many amino acids (from 3 to 6) and displays the best ΔG_Binding_ values, remaining constant during the MD simulations. Similar observations for all ligands showed consistency with the docking studies. At least one residue of the catalytic dyad is always involved in H-bond interactions with all 5 ligands over the MD simulation duration, in particular Cys145 for CID 1632360, CID 645492, CID 2193552, and His41 for CID 2193552. The persistence of the H-bonds over the MD simulation duration can be estimated by observing which and when the concerned residues enter or leave the H-bonding network (Table 4, Figure 7).

### 3.4. ADMET Studies

The ADMET properties of the nine (four covalent and five non-covalent) potential inhibitors, predicted using SwissADME [49], pkCSM [50], and ProTox-II [51], are listed in Table 5. Regarding bioavailability, all nine molecules respect Lipinski’s “rule of five” [52] and, partially, Veber’s rule [53]. All identified derivatives except CID 2193552 have very good predicted overall intestinal absorption, with high Caco-2 permeability (log Papp > 0.9) and intestinal absorption over 90%. Although the predicted steady-state volume of distribution (VDss) and the fraction unbound to serum proteins are both low, the compounds seem unlikely to cross the blood-brain barrier (BBB) and reach the central nervous system (CNS). Most compounds are also not likely to be metabolized by the two main isoforms of cytochrome P450. Finally, almost no compound showed predicted hepato-, immuno-, or cytotoxicity. Anticipated carcinogenicity could be observed for three covalent and three non-covalent potential inhibitors). Overall, the nine compounds exhibit satisfactory predicted pharmacokinetic properties, adequate for being considered as potential drug candidates for COVID-19 infection treatment.

## 4. Conclusions

The investigation of the homology between SARS-CoV-2 and the avian IBV main proteases has revealed a similar overall secondary structure, a fully conserved His41 and Cys145 catalytic dyad, and some differences within other residues of their active sites, justifying the search for SARS-CoV-2 M^Pro^ among reported IBV 3CLPro inhibitors. A library of bioactive substances on the avian IBV protease (PubChem BioAssay AID 1706) was thus used to look for covalent and non-covalent inhibitors by docking studies within the active site of SARS-CoV-2 M^Pro^. Four covalent candidate inhibitors (including chloroacetyl derivatives, acrylamides, benzonitriles, nitriles, and 2-cyanoacetamides) and five non-covalent (hydrazine, thiazole, benzotriazole, and diaminophenyl derivatives) potential inhibitors have been identified and their binding modes investigated. The covalent ones interact tightly with the active site through several H-bonds and locate their electrophilic function at the reactive distance of the SH group of Cys145. The non-covalent potential inhibitors also bind tightly with the active site residues and are located in close proximity to the catalytic dyad. Molecular dynamics simulations confirmed that these ligands remain bound to the active site of the protein. Protein-ligand binding free energies appear consistent with the docking studies and highlight the residues responsible for most of the binding, notably the catalytic dyad, which develops key interactions with all identified ligands. Further, ADMET predictions showed calculated pharmacokinetic properties that are consistent with possible use as drug candidates. Overall, this bioinformatics study contributes to the identification of new bioactive compounds with possible activity against COVID-19.

## Figures and Tables

**Table 1:** 2D and 3D binding modes of the four potential covalent inhibitors within the SARS-CoV-2 M^Pro^ active site (Cys145 is indicated in magenta in the 3D representation).

Compound CID— H-Bonds and Interactions with Amino Acids	Binding Modes
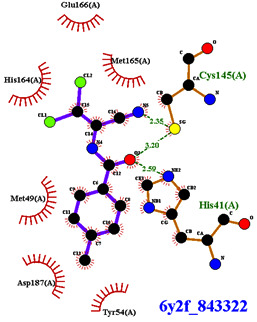	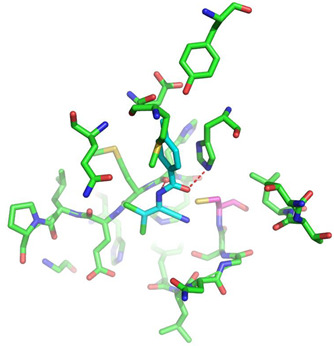
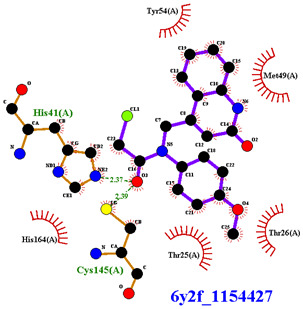	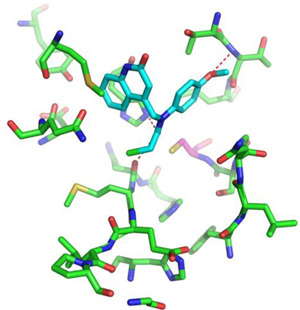
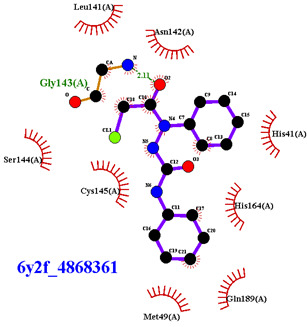	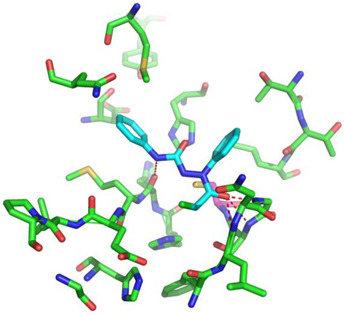
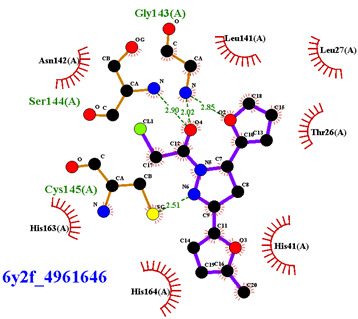	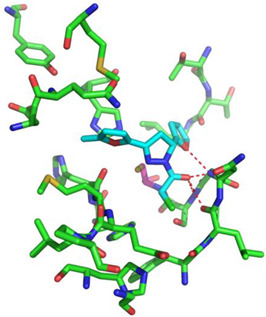

**Table 2:** Binding modes within the SARS-CoV-2 M^Pro^ active site of compounds ranked in the top 5, shown as the results of docking experiments for 388 out of the 405 active compounds on the avian IBV protease.

Compound CID— H-Bonds and Interactions with Amino Acids	Binding Modes and Docking Score (kcal/mol)
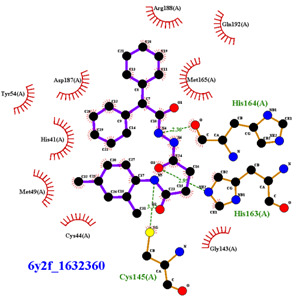	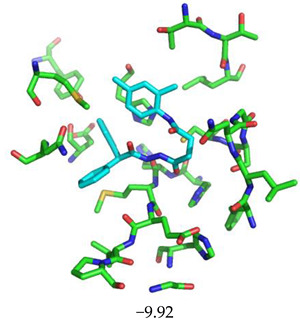
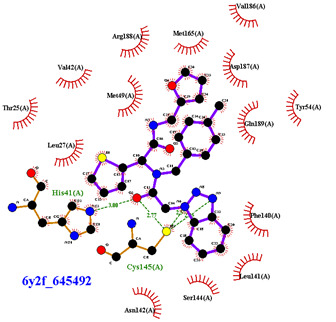	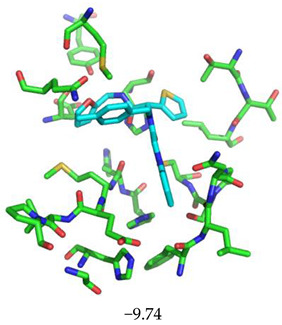
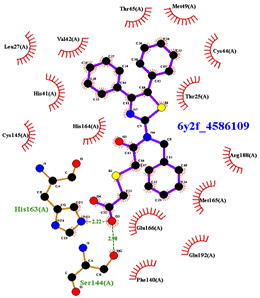	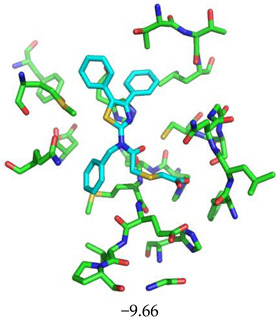
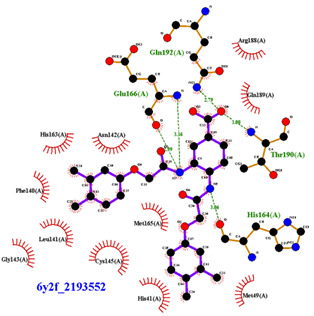	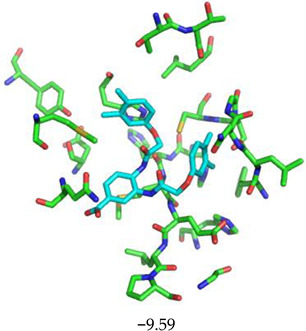
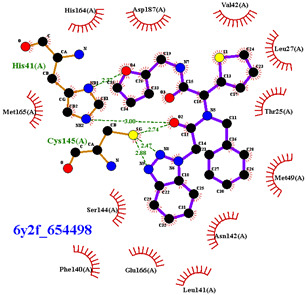	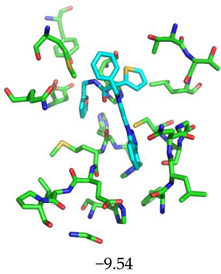

**Table 3:** Average ligands RMSD with hydrogen atoms and identification of important amino acids bound to the ligands via hydrogen bonds.

Compounds CID	Ligand RMSD (Å)	Residues Interacting via H-Bonds during the MD Simulations
1632360	1.602	Asn142, Gly143, Glu166
645492	1.087	Met49, Ser144, Gln189
2193552	1.851	His41, Met49, Asn51, Glu166, Asp187, Thr190, Gln192, Gln189
4586109	3.344	Gly143, Ser144, His163, Glu166, Gln189,
654498	1.717	Met49, Glu166, Gln189,

**Table 4:** ΔG_Binding_ (kcal/mol) of complexes collected each 1000 ps (residues involved in hydrogen bonds are indicated in brackets).

Compounds CID	1632360	645492	2193552	4586109	654498
ΔG_Binding_ t = 0	−9.16 (Gly143)	−9.21 (Gln189, Met49, Cys145)	−10.23 (Glu166, Thr190, Gln192)	−9.42 (Gly143, Ser144, His163, Glu166)	−8.52 (Met49, Gln189)
ΔG_Binding_ t = 1000	−9.34 (Gly143, Glu166)	−9.24 (Gln189)	−9.95 (His41, Glu166, Asp187, Gln189, Thr190, Gln192)	−9.91 (Gly143, Ser144, His163, Gln189)	−9.08 (Met49, Glu166, Gln189)
ΔG_Binding_ t = 2000	−9.47 (Gly143)	−9.08 (Gln189)	−10.28 (His41, Glu166, Asp187, Gln189, Thr190, Gln192)	−9.89 (Gly143, Ser144, His163, Gln189)	−9.17 (Cys145)
ΔG_Binding_ t = 3000	−9.37 (Asn142)	−9.28 (Met49)	−10.33 (His41, Glu166, Gln189, Thr190, Gln192)	−10.20 (Gly143, Ser144, His163, Gln189)	−8.70 (Glu166, Gln189)
ΔG_Binding_ t = 4000	−9.16 (Cys145)	−9.09 (Cys145)	−10.63 (Met49 Glu166, Thr190, Gln192)	−9.79 (Gly143 Ser144, Cys145, His163)	−9.05 (Met49, Glu166, Gln189)
ΔG_Binding_ t = 5000	−9.12 (Glu166)	−9.22 (Ser144)	−10.52 (Met49, Asn51, Glu166, Thr190, Gln192)	−9.82 (Gly143 Ser144, His163, Gln189)	−9.36 (Glu166, Gln189)

**Table 5:** Predicted ADMET properties for identified potential M^Pro^ inhibitors.

Property	Unit	CID 843322	CID 1154427	CID 4868361	CID 4961646	CID 1632360	CID 645492	CID 2193552	CID 4586109	CID 654498
Molecular weight	g/mol	255.10	356.80	303.74	292.72	429.51	499.58	476.52	474.59	485.56
LogP		2.55	2.92	2.32	2.27	3.70	3.60	3.92	4.89	3.22
H-bond donors		1	1	2	0	3	1	3	1	1
H-bond acceptors		2	3	2	4	3	5	6	4	5
Rotatable bonds		3	6	7	4	11	11	11	10	11
PSA	Å^2^	52.89	62.40	61.44	58.95	87.30	121.50	113.96	124.04	121.50
Caco_2_ permeability	log Papp in 10–6 cm/s	1.324	1.133	1.307	1.315	0.793	0.739	0.626	1.04	0.83
Intestinal absorption (human)	% Absorbed	91.937	96.64	91.279	96.937	93.663	94.393	70.344	93.421	93.919
VDss (human)	log L/kg	0.011	−0.076	−0.021	−0.21	−0.24	0.166	−1.842	−0.714	0.117
Fraction unbound (human)	Fu	0.319	0.038	0	0.34	0.024	0.086	0	0.278	0.072
BBB permeability	log BB	0.079	−0.077	0.244	0.122	−0.662	−0.429	−1.359	−0.996	−0.435
CNS permeability	log PS	−2.781	−2.35	−2.088	−2.877	−2.191	−2.239	−2.896	−2.257	−2.308
CYP2D6 inhibitor	Yes/No	No	No	No	No	No	No	No	No	No
CYP3A4 inhibitor	Yes/No	No	No	No	No	Yes	Yes	No	No	Yes
Total Clearance	log ml/min/kg	−0.054	0.086	−0.069	0.189	0.241	0.035	−0.045	0.624	0.091
Hepatotoxicity	Yes/No (probability %)	No (61)	No (71)	No (54)	Yes (51)	No (59)	No (58)	No (75)	No (57)	No (58)
Carcinogenicity	Yes/No (probability %)	No (59)	Yes (52)	Yes (66)	Yes (66)	Yes (56)	Yes (52)	No (69)	No (53)	Yes (52)
Immunotoxicity	Yes/No (probability %)	No (99)	Yes (87)	No (99)	No (98)	No (99)	No (99)	No (99)	No (99)	No (99)
Mutagenicity	Yes/No (probability %)	Yes (55)	Yes (52)	No (55)	No (57)	No (52)	Yes (55)	No (79)	No (65)	Yes (56)
Cytotoxicity	Yes/No (probability %)	No (76)	Yes (57)	No (72)	No (68)	No (77)	No (61)	No (60)	No (66)	No (63)

## Data Availability

Data can be found in the manuscript and in the Supplementary Materials.

## Supplementary Materials

The following supporting information can be downloaded at: https://www.mdpi.com/article/10.3390/biom13060956/s1, Figure S1. Docking experiments using a genetic algorithm. To corroborate the binding modes obtained using the Argusdock engine (in cyan color), docking experiments with a genetic algorithm engine implemented in Arguslab was achieved leading to the binding modes in magenta color. As depicted in the different figures, the binding modes obtained with the two methods are consistent for all compounds with only some differences for the compound 843322. A. Covalent inhibitors. B. Non-covalent inhibitors; Figure S2. Calpha RMSD obtained by molecular dynamics simulations during 500 ps for the protein without ligand and for all reversible complexes. Molecular dynamics simulations were performed for 500 ps in triplicate to ascertain that the complexes between the protein and the potential reversible inhibitors were stable. It could be noted that the different compounds remain tightly bound to the active site during the simulation; Table S1. Compound CID of the 40 potential covalent inhibitors; Table S2. Summary of results in order of docking score (kcal/mol).
