# Supplementary material for: In Silico Identification of Potential Inhibitors of the SARS-CoV-2 Main Protease among a PubChem Database of Avian Infectious Bronchitis Virus 3CLPro Inhibitors
Source: Biomolecules. 2023 Jun 7;13(6):956. doi: 10.3390/biom13060956 (PMC10296608; doi:10.3390/biom13060956)
Supplement: Supplementary file 1 [file biomolecules-13-00956-s001.zip › biomolecules-2394443-supplementary.pdf]

In silico identification of potential inhibitors of the SARS-CoV-2 Main protease among a PubChem database of avian infectious bronchitis virus 3CLPro inhibitors

Laurent Soulère[1]*, Thibaut Barbier[1] and Yves Queneau[1]

[1] Univ Lyon, INSA Lyon, Université Claude Bernard Lyon 1, CPE Lyon, UMR 5246, CNRS, ICBMS, Institut de Chimie et de Biochimie Moléculaires et Supramoléculaires, Bât. E. Lederer, 1 rue Victor Grignard F-69622 Villeurbanne, France.

*Corresponding author Laurent Soulère (laurent.soulere@insa-lyon.fr)

Supplementary information

Table S1. Compound CID of the 40 potential covalent inhibitors.

135403580	23844236	7314640	6903089
5928454	5918347	5735038	5734448
4961648	4961646	4868361	4437603
4381126	3912197	3859741	3811656
3735802	3686662	3592691	3555173
3473830	3192987	3162837	2526861
2454965	2440506	2416356	2412504
2134372	2104090	2064069	2017227
1537038	1177253	1154427	859620
843322	738894	660907	625593

Table S2. Summary of results in order of docking score (kcal/mol).

1. 1632360 -9.92	126. 3192987 -8.20434	259. 2890716 -7.53104
2. 645492 -9.73855	127. 9566958 -8.20273	260. 2815694 -7.52643
3. 4586109 -9.66179	128. 3206178 -8.19011	261. 4868361 -7.51938
4. 2193552 -9.58966	129. 16825531 -8.17704	262. 24789625 -7.51891
5. 654498 -9.53933	130. 2853037 -8.16736	263. 3878400 -7.50038
6. 2106192 -9.51193	131. 135845989 -8.15589	264. 1069254 -7.49395
7. 2734449 -9.50301	132. 2452051 -8.15212	265. 24820042 -7.47138
8. 649868 -9.49126	133. 5399054 -8.13972	266. 3162837 -7.47007
9. 24789769 -9.47309	134. 9596269 -8.13802	267. 2497380 -7.46669
10. 2898209 -9.46584	135. 5824720 -8.13435	268. 4944777 -7.46307
11. 3191728 -9.44825	136. 677475 -8.12396	269. 4418 -7.4629
12. 652480 -9.36413	137. 2017227 -8.10258	270. 799395 -7.46181
13. 1250233 -9.27835	138. 2108058 -8.10089	271. 4395364 -7.46143
14. 1007780 -9.20512	139. 2993330 -8.10087	272. 676085 -7.45824
15. 3178307 -9.17304	140. 3240186 -8.0951	273. 2814665 -7.45753
16. 970829 -9.13668	141. 3128209 -8.08638	274. 2348921 -7.45184
17. 3129448 -9.11774	142. 16007641 -8.08004	275. 3196508 -7.44268
18. 893627 -9.0865	143. 24790237 -8.07894	276. 6460643 -7.43278
19. 1327906 -9.06878	144. 2870407 -8.07892	277. 3555173 -7.42276
20. 3197965 -9.06648	145. 807412 -8.07041	278. 4438422 -7.42077
21. 2385601 -9.06124	146. 1117371 -8.06726	279. 4900532 -7.40898
22. 24817257 -9.0573	147. 2057118 -8.06577	280. 2449860 -7.4037
23. 12005302 -9.04904	148. 661510 -8.06089	281. 5713900 -7.39864
24. 951610 -9.04096	149. 6023693 -8.05721	282. 3237441 -7.39671
25. 648736 -9.0309	150. 2815268 -8.05629	283. 24819855 -7.39349
26. 3929515 -9.02983	151. 1304219 -8.05366	284. 9563422 -7.38664
27. 3475954 -9.02289	152. 6880919 -8.05235	285. 3293421 -7.37769
28. 2965851 -9.0223	153. 2974727 -8.0511	286. 2815848 -7.37555
29. 3198832 -8.99767	154. 2417657 -8.04805	287. 2472898 -7.37539
30. 1420934 -8.9706	155. 3962842 -8.04607	288. 2416356 -7.37334
31. 3202425 -8.9593	156. 3235672 -8.03114	289. 6465235 -7.36997
32. 1768585 -8.95878	157. 1189783 -8.03083	290. 1975036 -7.36272
33. 24789497 -8.95446	158. 660907 -8.02645	291. 2917531 -7.35955
34. 2356903 -8.9378	163. 1010988 -8.00793	292. 722579 -7.34384
35. 4181547 -8.91597	164. 24790050 -7.9995	293. 2813012 -7.3399
36. 2925127 -8.91187	165. 826461 -7.99788	294. 5918347 -7.33494

37. 2829992 -8.91049	166. 4371580 -7.99744	295. 3110581 -7.3256
38. 2928698 -8.89488	167. 24820087 -7.99533	296. 24790041 -7.31994
39. 16017527 -8.88818	168. 24817255 -7.99471	297. 5897880 -7.28826
40. 16812389 -8.8826	169. 135403580 -7.98943	298. 930620 -7.28801
41. 644611 -8.86571	170. 4381126 -7.97039	299. 1936560 -7.28217
42. 5114648 -8.85093	171. 2915854 -7.96382	300. 2440506 -7.27198
43. 4187185 -8.84409	172. 2971444 -7.96192	301. 765284 -7.26409
44. 2410199 -8.83455	173. 1008368 -7.96162	302. 3686662 -7.26236
45. 16034541 -8.8304	174. 4679126 -7.95729	303. 4961646 -7.25739
46. 3221960 -8.81198	175. 1160939 -7.95248	304. 2739954 -7.25624
47. 135531840 -8.78196	176. 741948 -7.93507	305. 4220289 -7.25432
48. 3590295 -8.77158	177. 4189659 -7.93463	306. 675749 -7.25253
49. 6236746 -8.76236	178. 2458897 -7.93165	307. 805223 -7.24918
50. 5717 -8.75468	179. 2870396 -7.92167	308. 710241 -7.24417
51. 22551764 -8.74868	180. 4910360 -7.92089	309. 972394 -7.24199
52. 135497987 -8.74468	181. 2064069 -7.91399	310. 9661081 -7.23867
53. 655942 -8.72583	182. 3992501 -7.90199	311. 2406331 -7.22918
54. 778154 -8.71022	183. 6903089 -7.89867	312. 1249482 -7.22681
55. 1295693 -8.70192	184. 9557232 -7.89733	313. 135484279 -7.22036
56. 649398 -8.67937	185. 2808586 -7.89542	314. 16404473 -7.20407
57. 9563955 -8.67512	186. 9585555 -7.89447	315. 1154427 -7.20204
58. 3196510 -8.6511	187. 3237586 -7.88875	316. 2310813 -7.19879
59. 2301119 -8.64617	188. 2323124 -7.88743	317. 3158067 -7.19735
60. 3091264 -8.64133	189. 2526861 -7.87817	318. 2102546 -7.18544
61. 3191638 -8.63335	190. 1214257 -7.87137	319. 644661 -7.18237
62. 24819986 -8.62912	191. 4447969 -7.86238	320. 2083510 -7.17396
63. 859620 -8.62852	192. 1224165 -7.85769	321. 1092464 -7.16823
64. 1831132 -8.62295	193. 1907744 -7.85036	322. 5928454 -7.15587
65. 1295791 -8.61462	194. 9549963 -7.84345	323. 2078229 -7.14222
66. 1312344 -8.60582	195. 2997527 -7.84222	324. 3242436 -7.13751
67. 664943 -8.60251	196. 887990 -7.83157	325. 4836362 -7.12886
68. 4907806 -8.55872	197. 4330749 -7.82774	326. 3794723 -7.12659
69. 24790407 -8.54843	198. 728528 -7.82154	327. 986562 -7.11671
70. 6904494 -8.54456	199. 3191502 -7.82036	328. 740914 -7.1036
71. 2558401 -8.54403	200. 7254819 -7.81794	329. 2998767 -7.09836
72. 6470683 -8.54183	201. 859639 -7.81734	330. 211186 -7.07389
73. 645365 -8.54119	202. 2282752 -7.81668	331. 4621376 -7.05822
74. 2849510 -8.53732	203. 930290 -7.81656	332. 24817263 -7.05523
75. 2966254 -8.52518	204. 3245569 -7.81166	333. 3640363 -7.05073
76. 3196509 -8.52323	205. 9566932 -7.80815	334. 880528 -7.04625
77. 3152897 -8.52246	206. 7314640 -7.80713	335. 5741945 -7.03546
78. 3774350 -8.51215	207. 2134372 -7.80655	336. 4437603 -7.00567
79. 4175307 -8.50259	208. 135693201 -7.80403	337. 1299520 -7.00428
80. 135684269 -8.48387	209. 16017324 -7.79686	338. 2963113 -7.00305
81. 5756577 -8.46296	210. 2104090 -7.79091	339. 17582529 -7.00268
82. 2121076 -8.46175	211. 16019819 -7.78421	340. 1120962 -6.9986
83. 5735038 -8.44614	212. 4182036 -7.78306	341. 843322 -6.99648
84. 73157 -8.43978	213. 2898168 -7.78222	342. 4961648 -6.99184
85. 24817254 -8.43505	214. 4542341 -7.77263	343. 2333509 -6.96988
86. 615593 -8.43126	215. 649992 -7.77205	344. 87247 -6.9553
87. 738894 -8.42879	216. 5824722 -7.76972	345. 2294766 -6.94213

88. 5302421 -8.41654	217. 20903731 -7.76884	346. 2147111 -6.92723
89. 2949086 -8.3926	218. 3473830 -7.76858	347. 17584476 -6.92627
90. 1891999 -8.39107	219. 1083882 -7.76511	348. 2845421 -6.90861
91. 648374 -8.38488	220. 597363 -7.76367	349. 22515899 -6.89952
92. 3206295 -8.37375	221. 22518136 -7.75643	350. 2454965 -6.87649
93. 2396278 -8.3701	222. 2561190 -7.75263	351. 17580184 -6.85425
94. 2870196 -8.35766	223. 20862125 -7.75103	352. 4377849 -6.84425
95. 3037 -8.35016	224. 659929 -7.74981	353. 22583081 -6.80264
96. 5734490 -8.34672	225. 3592691 -7.74671	354. 17583883 -6.80204
97. 22517479 -8.34412	226. 708625 -7.73773	355. 16807543 -6.77372
98. 3191469 -8.34175	227. 4917203 -7.73736	356. 9637339 -6.76054
99. 3257857 -8.33959	228. 2078638 -7.73099	357. 1188014 -6.75284
100. 3239069 -8.33664	229. 1121490 -7.72921	358. 1537038 -6.75275
101. 1177253 -8.33521	230. 3198732 -7.72783	359. 601670 -6.73867
102. 3244307 -8.33007	231. 2999977 -7.71084	360. 736374 -6.73149
103. 1475477 -8.3279	232. 9550250 -7.70815	361. 215090 -6.72578
104. 2514618 -8.32352	233. 3912197 -7.70573	362. 647501 -6.70801
105. 3211933 -8.32109	234. 657485 -7.6997	363. 948801 -6.68971
106. 135451179 -8.31321	235. 2412504 -7.69793	364. 5509654 -6.67146
107. 2229132 -8.30873	236. 9611292 -7.69345	365. 460749 -6.64892
108. 1864299 -8.30855	237. 638860 -7.6886	366. 6403417 -6.62583
109. 135472652 -8.30565	238. 24791295 -7.68562	367. 6470895 -6.57876
110. 6268409 -8.30147	239. 9551063 -7.68471	368. 4794522 -6.57814
111. 2814640 -8.29469	240. 666722 -7.68222	369. 3811656 -6.55464
112. 17379494 -8.29127	241. 2320755 -7.6689	370. 900806 -6.51362
113. 5734448 -8.285	242. 3634352 -7.66201	371. 906542 -6.5112
114. 651774 -8.27142	243. 665102 -7.65172	372. 3859741 -6.37181
115. 5341357 -8.25559	244. 2083279 -7.62931	373. 6246323 -6.22226
116. 3735802 -8.25503	245. 1105927 -7.62636	374. 4137407 -6.1767
117. 2515806 -8.25437	246. 1949127 -7.61699	375. 2057165 -6.1189
118. 2352096 -8.25242	247. 4911266 -7.60034	2443805, no acceptable poses
119. 2939288 -8.25107	248. 54688584 -7.59864	135435911, no acceptable poses
120. 761840 -8.23951	249. 705256 -7.59722	54677920, no acceptable poses
121. 2420336 -8.23546	250. 728514 -7.58977	24789460, no acceptable poses
122. 9662819 -8.23421	251. 2530053 -7.58557	16195219, no acceptable poses
123. 9643441 -8.23392	252. 24761221 -7.5821	9666324, no acceptable poses
124. 7996726 -8.22261	253. 2966450 -7.57932	9636511, no acceptable poses
125. 6225958 -8.2069	254. 2149567 -7.57492	9609431, no acceptable poses
159. 2097351 -8.02097	255. 5349620 -7.57175	2958977, no acceptable poses
160. 2384236 -8.01443	256. 2145491 -7.56374	1341336, no acceptable poses
161. 4077726 -8.01364	257. 6878045 -7.55574	1295575, no acceptable poses
162. 24791306 -8.01078	258. 2951459 -7.53473	1009382, no acceptable poses

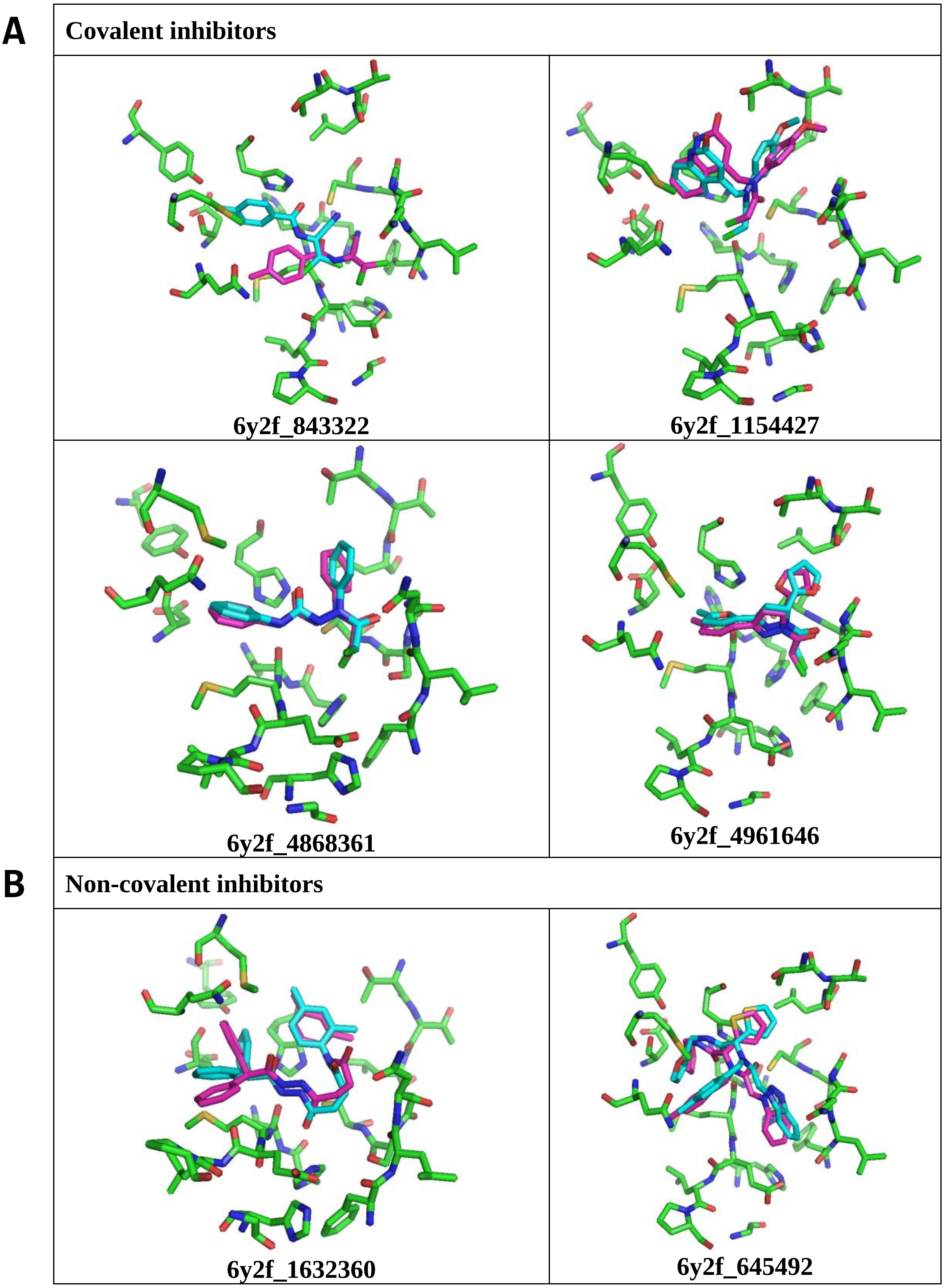
A
Covalent inhibitors
6y2f_843322
6y2f_1154427
6y2f_4868361
6y2f_4961646
B
Non-covalent inhibitors
6y2f_1632360
6y2f_645492

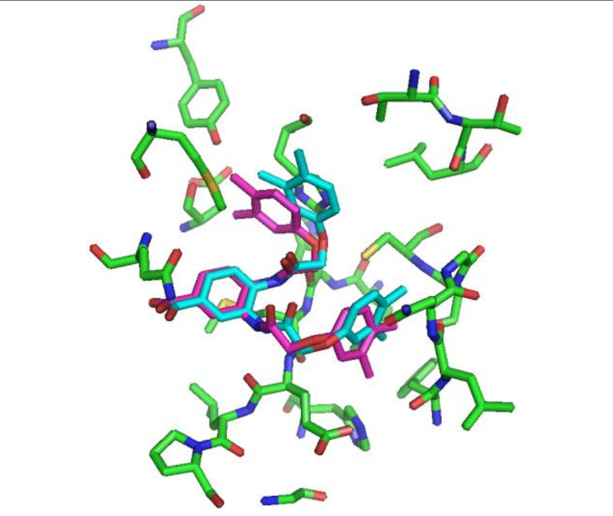

6y2f_2193552

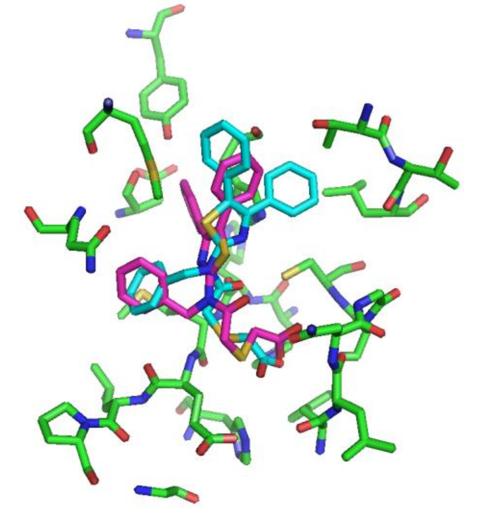

6y2f_4586109

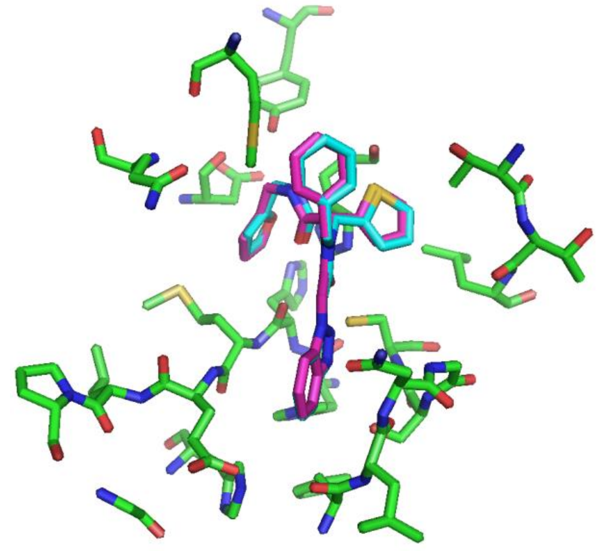

6y2f_654498

Figure S1. Docking experiments using a genetic algorithm. To corroborate the binding modes obtained using the Argusdock engine (in cyan color), docking experiments with a genetic algorithm engine implemented in Arguslab was achieved leading to the binding modes in magenta color. As depicted in the different figures, the binding modes obtained with the two methods are consistent for all compounds with only some differences for the compound **843322**. **A.** Covalent inhibitors. **B.** Non-covalent inhibitors.

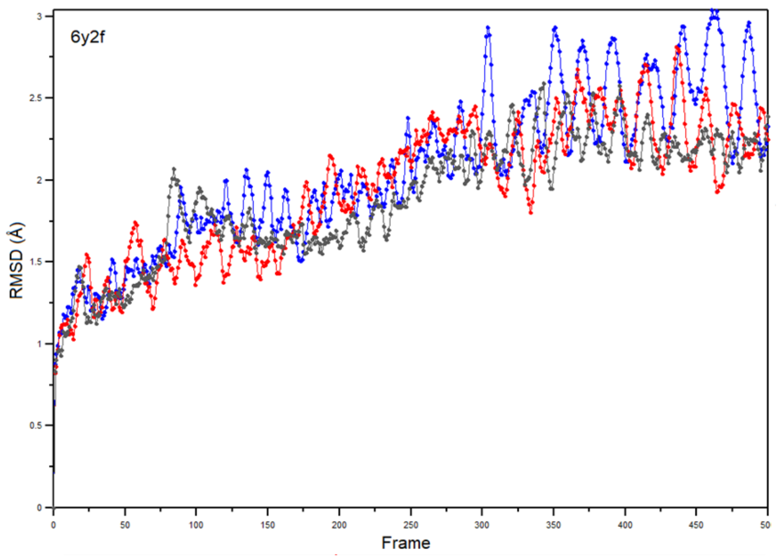
6y2f
RMSD (Å)
0
0.5
1
1.5
2
2.5
3
0
50
100
150
200
250
300
350
400
450
50
Frame

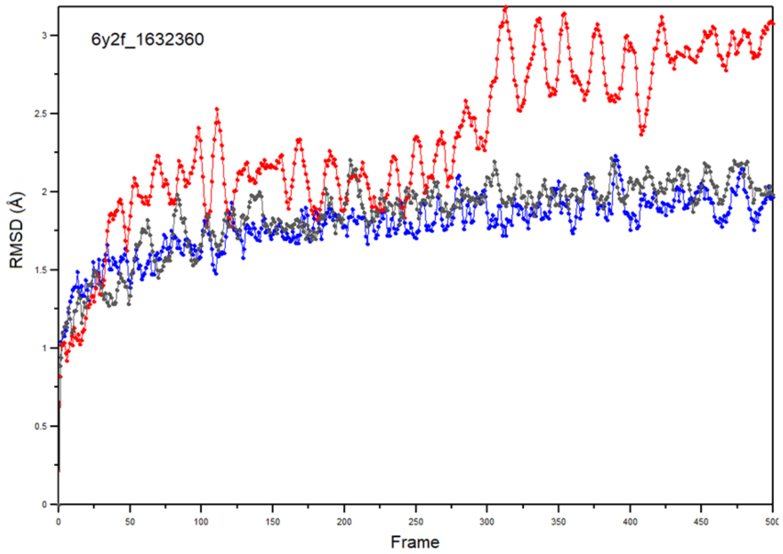
6y2f_1632360
RMSD (Å)
0
0.5
1
1.5
2
2.5
3
0
50
100
150
200
250
300
350
400
450
50(
Frame

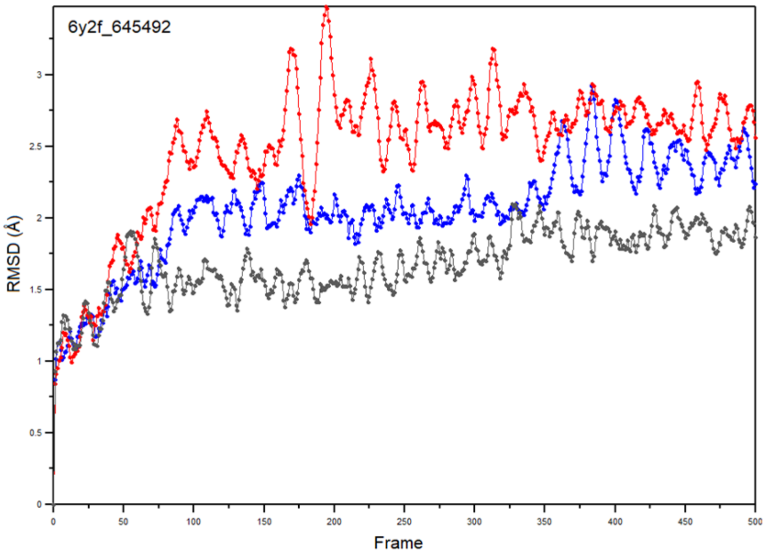
6y2f_645492
RMSD (Å)
0
0.5
1
1.5
2
2.5
3
0
50
100
150
200
250
300
350
400
450
500
Frame

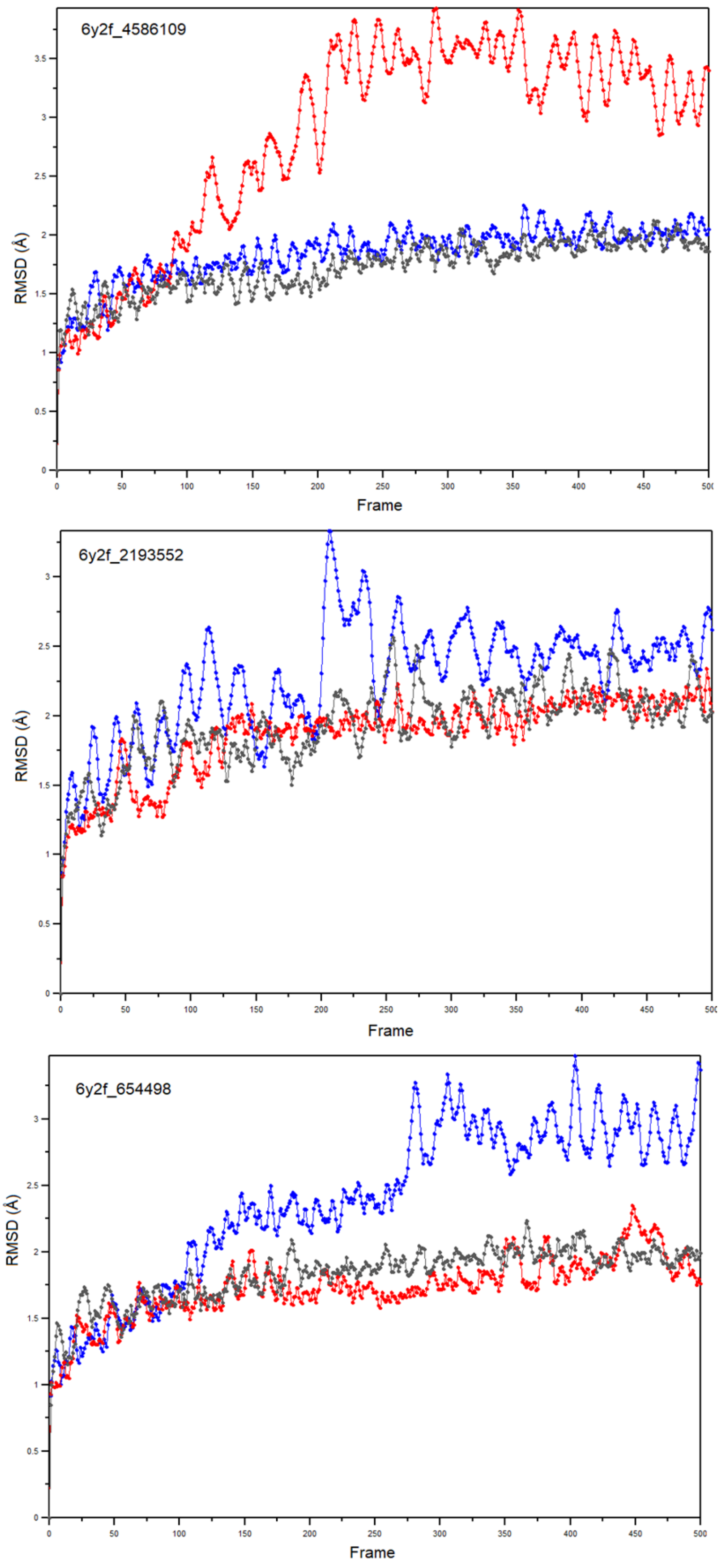

Figure S2. Calpha RMSD obtained by molecular dynamics simulations during 500 ps for the protein without ligand and for all reversible complexes. Molecular dynamics simulations were performed for 500 ps in triplicate to ascertain that the complexes between the protein and the potential reversible inhibitors were stable. It could be noted that the different compounds remain tightly bound to the active site during the simulation.
